# Tensile Strength of Novel Nonabsorbable PTFE (Teflon®) versus Other Suture Materials: An In Vitro Study

**DOI:** 10.1155/2019/7419708

**Published:** 2019-10-09

**Authors:** José Arce, Alondra Palacios, Daniel Alvítez-Temoche, G. Mendoza-Azpur, Percy Romero-Tapia, Frank Mayta-Tovalino

**Affiliations:** ^1^Faculty of Stomatology, Universidad Peruana Cayetano Heredia, Lima, Peru; ^2^Faculty of Dentistry, Universidad Nacional Federico Villarreal, Lima, Peru; ^3^Department of Periodontology, School of Dentistry, Universidad Científica del Sur, Lima, Peru

## Abstract

**Objective:**

To compare the in vitro tensile strength of sutures used in implant surgery according to the type of thread and the immersion time in artificial saliva.

**Methods:**

For the development of the study, three suture materials were used: polyglactin 910 (PG), black silk (BS), and Teflon (PTFE) 4-0; 150 samples were used, which were divided among each type of suture and then subdivided into five groups of 10 according to the various immersion times (baseline, 3, 7, 14, and 21 days) in artificial saliva. A universal test machine was used to measure the tensile strength at a speed of 25 cm/min, stretch each sample until the material fails, and record the maximum strength in Newtons (N). Finally, the failure point of the samples was evaluated at 10× increase using a stereromicroscope (Leica Biosystems).

**Results:**

When analyzing the tensile strength of the various groups of sutures, it was evidenced that PG maintained its strength, which was lowest at baseline and highest at 21 days. When performing the statistical inference of PG and PTFE, it was found that the force necessary to achieve detachment was not statistically significant (*p* < 0.05). However, it was shown that the force necessary to achieve rupture in the BS group was statistically significant (*p*=0.001).

**Conclusion:**

To sum up, when comparing the in vitro tensile strength of PG, BS, and PTFE sutures at baseline and 3, 7, 14, and 21 days, there was no statistically significant difference. This indicates that all sutures used present sufficient performance that remains resistant as time progresses.

## 1. Introduction

With the advent of technology, the number of suture material options in surgeries has increased rapidly [1–5]. New suture materials are being continuously developed and made available in the market. However, the choice of a type of suture for the fixation of soft tissues requires certain considerations: (a) the fixation strength must provide an adequate approximation of the tissues during the critical period of healing; (b) the size of knots and sutures must be within acceptable limits; and (c) the inflammatory reaction of the tissues to the suture should be minimal.

The fixing strength of a suture is basically due to its ability to approximate soft tissues, and this depends on the mechanical properties of the technique and the suture material used. The tensile properties, tensile strength, elasticity, and biocompatibility of the suture material are some of the factors that determine the performance of the suture during clinical use [3]. A clear example is the development of biodegradable sutures, such as polymers or polyglycolic acid, which have provided surgeons with a suture that causes a minimal tissue reaction [6–9]. However, the tensile strength of these suture materials decreases during the duration of immersion in a saline solution, which is a possible limitation that encourages the search for an ideal suture material.

Suturing is performed for various purposes in the surgical field, such as the primary closure of tissues that were separated during a surgical implant procedure or accidental trauma, and it promotes healing and manages bleeding [10]. The materials used for suturing consist of sutures, tissue adhesives, and staplers; these materials are collectively referred to as suture materials. Among these, the suture is the most commonly used material. The ideal suture material must meet several requirements, such as [11] good tensile strengths, easy to handle, and able to use to form secure knots. Additionally, because sutures must be biologically inert, they must induce minimal inflammation of the tissues, and they cannot promote infection.

Scientific evidence indicates that the resistance and adhesion of the sutured tissue increases with time, and a significant increase in flap resistance is achieved between 1 week and 2 weeks after surgery. Therefore, a deficiency in the resistance of the suture material can cause premature rupture of the suture, which leads to poor adaptation of the surgical flaps and the induction of tissue healing by second intention [12]. Tensile strength is a characteristic that needs to be maintained because suture material tends to lose between 70 and 80% of its initial strength. Therefore, the necessary initial tensile strength in a horizontal plane must be guaranteed to avoid breaking the suture material [13].

Thus, this study compared the tensile strength of one absorbable and two nonabsorbable suture materials, namely, PG: polyglactin, BS: black silk, and PTFE: polytetrafluoroethylene (Teflon), according to different immersion times in artificial saliva.

## 2. Materials and Methods

### 2.1. Sample Size

In the present *in vitro* experimental study, black silk, Teflon, and polyglactin sutures were used; they were submerged in artificial saliva for various durations. As mentioned, the sample size was determined based on the pilot test by means of a comparison formula using Stata 12.0 software (*n* = 10). For the calculation of the sample, an alpha error of 0.05 and a power of 0.80 were used. Then, the specimens were divided between each type of suture and then subdivided into five groups of 10 according to the different immersion times (baseline, 3, 7, 14, and 21 days) in artificial saliva correspondingly. Finally, a universal testing machine was used to measure tensile strength in Newtons.

### 2.2. Suture Types

Three suture materials were used: Teflon monofilament (PTFE) (Omnia®; Fidenza; Parma, Italy) nonabsorbable suture, nonabsorbable multifilament black braided silk sutures (Unilene Peruvian Surgery; Napo 450; Lima, Peru), and polyglactin 910 multifilament absorbable sutures (Vicryl®; Ethicon, Brazil); all specimens were USP 4-0 caliber.

### 2.3. Composition of Synthetic Saliva

The Salival® product is a colorless, viscous substance composed of Na^+^, K^+^, and CL^+^ at a percentage ratio in relation to the purified aqueous volume. It has a pH and viscosity similar to natural saliva (Table 1).

### 2.4. Biological Simulation

An *in vitro* biological simulation of the oral environment was generated by mixing 2 ml of sterile artificial saliva and serum at a 1 : 1 ratio in a Petri dish. This biological solution was prepared and maintained at a pH in the range from 7.4 to 8.1, similar to natural saliva, at room temperature (17°C to 20°C). The samples were placed in a 4 ml plastic container containing the mixed serum and artificial saliva (Salival®) to simulate the oral environment; the samples were labeled and maintained for a certain time in a state without tension [14, 15].

### 2.5. Tensile Strength Test

**Figure 1:**
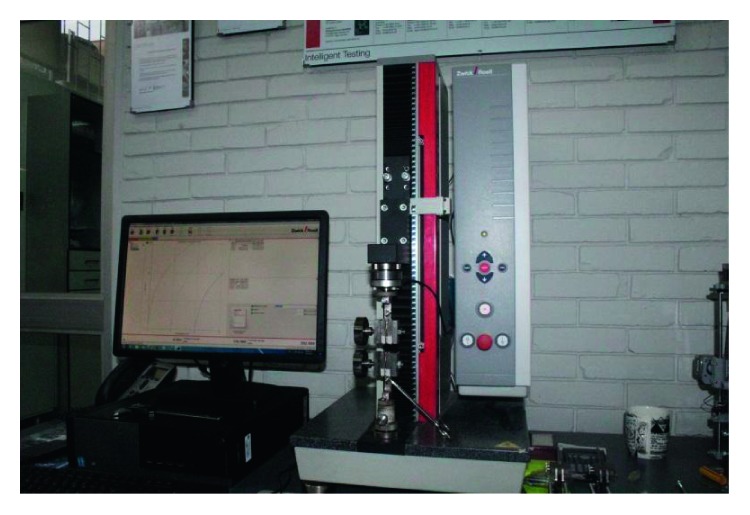
Material testing machine BT1-FR0.5TS.D14 (Zwick GmbH & Co.KG), with which the tensile measurements of all suture materials evaluated were performed.

Each sample was prepared with a knot around two metal poles installed in the universal testing machine with a fixed distance of 15.0 mm between the two poles. The tensile strengths of the suture samples were tested at specific times: preimmersion (baseline) and postimmersion at 3, 7, 14, and 21 days. The evaluation of the tensile strength of the suture samples was performed using a Material Testing Machine (BT1-FR0.5TS.D14; Zwick GmbH & Co.KG; August-Nagel-Strabe 1189079; Ulm Germany; manufactured in 2014) at a speed of 25 cm/min. Each sample was stretched until the material failed, and the maximum load was recorded in Newtons (N) (Figure 1) and tabulated for analysis. The break points of the samples were evaluated at 10× magnification using a Leica stereomicroscope [14, 15].

### 2.6. Statistical Analysis

The statistical analysis was performed with the Stata 12.0 software. First, the univariate analysis was carried out by measuring the central tendency (arithmetic mean) and the measure of dispersion (standard deviation) of the variable tensile strength of the suture materials (PG, BS, and PTFE). The statistical assumptions of normality and homoscedasticity were then explored using the Shapiro–Wilk test and the Bartlett test. Finally, the bivariate analysis was performed using the two-way analysis of variance (ANOVA) and Kruskal–Wallis tests depending on the values obtained in the previous analysis. Finally, statistical significance was established with *p* < 0.05.

## 3. Results

### 3.1. Tensile Strength

**Figure 2:**
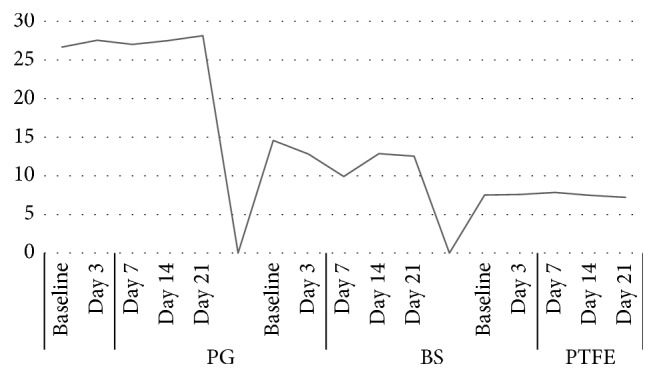
Tensile strength of various absorbable and nonabsorbable sutures according to immersion time in synthetic saliva. The tensile strength values are means expressed in Newtons. PG: polyglactin, BS: black silk, PTFE: polytetrafluoroethylene (Teflon).

When analyzing the descriptive statistics, it was found that in relation to the polyglactin (PG) group, the average tensile strength was stable for the various immersion times (baseline and days 3, 7, 14, and 21). The highest mean (28.14 ± 0.89 N) was found on the day (day 21). Therefore, when making the inference with the Kruskal–Wallis test, it was concluded that there were no statistically significant differences in tensile strength of the PG suture with respect to the different immersion times (*p*=0.522). Therefore, it is shown that this material remains stable over time despite immersion in artificial saliva (Table 2; Figure 2).

This phenomenon is not reflected in the black silk (BS) group, which showed heterogeneity in tensile strength. For example, the highest resistance was found at baseline 14.58 ± 1.64 N, and this progressively decreased in direct proportion to the time of immersion in artificial saliva. When performing, the inference was found that in this group, there were statistically significant differences between tensile strength and immersion time (*p*=0.001), which indicates that it is an unstable suture material in its resistance to tension over time (Table 2).

Table 2 shows that when evaluating the PTFE (Teflon) suture, a clear stability of tensile strength was found at various immersion times. In this group, no statistically significant differences were found (*p*=0.094). Therefore, this material shows excellent dimensional stability that, in addition to its uniform surface, makes it a material of choice for oral surgeries.

## 4. Discussion

The success of oral rehabilitation with implants depends on the osseointegration of the implant and the maintenance of the integrity of the perimplant soft tissues. When dental implants are placed, the surgical flap may change in position. The flap should be sutured to maintain its position and immobility. Therefore, soft tissue fixation techniques are important variables to consider in procedures [1–4]. With advancing technology, alternative fixation materials have increased rapidly. Therefore, the purpose of the study was to compare the *in vitro* tensile strength of sutures used in implant surgeries according to thread type and immersion time.

The pH of the medium is another important factor in the resorption of suture materials. Therefore, average pH was monitored every day and maintained between 7.4 and 8.1. The solution was replenished every 2 days when a variation in pH was observed, as a decrease in pH leads to resorption of the material. The duration of this study and the selection of test points were based on clinical relevance; the study was conducted for 3 weeks because the sutures for most surgical procedures in oral implantology are eliminated by that time.

In the present work, when analyzing the tensile strength of the sutures, polyglactin maintained strength: the lowest value was obtained at baseline, and the highest was obtained on day 21, for the dislocation of the suture. The comparison showed that the necessary force to achieve detachment was not statistically significant (*p*=0.522). In addition, the strength of Teflon decreased, with the lowest value on day 21 and the highest on day 7, for the dislocation of the suture. The comparison showed that the necessary force to achieve detachment was not statistically significant (*p*=0.094). However, the force necessary to displace black silk sutures decreased, with the lowest values observed on day 7 and the highest at baseline. The comparison found that the necessary force to achieve detachment was statistically significant (*p*=0.001).

In an evaluation of the tensile strength of surgical sutures, Kim et al. [16] used bioabsorbable sutures submerged in a saline solution for 14 days. They noted that tensile strength decreased significantly for chromic catgut sutures after that time; however, they did not assure whether the medium used had a significant effect on these findings. In contrast, in the present research, the Teflon and polyglactin sutures maintained their strength after 21 days of evaluation. This difference in approach could explain the variation in tensile strength between the studies.

Several studies on polyglactin [17–20] showed excellent handling properties, high initial tensile strength, and fewer tissue reactions. The multistrand specified that polyglactin 910 coated with PG 370 is intended to facilitate knotting and decrease tissue inflammation [21]. In the present work, Teflon and polyglactin sutures maintained most of their original strength over the 3-week study, which contradicts the conclusions of one study [22] that reported that polyglycolic acid sutures were more predictable and absorbed slowly in a wet environment; probably, because in *in vitro* studies, there is no influence of bacterial proteolytic enzymes, which may explain the different results found.

On the other hand, the monofilament and Polyglactin® resisted higher tensile forces compared with BS and PTFE, so it would be indicating that this seems to be more elastic. It should be noted that it is necessary to perform clinical studies to confirm these findings and thus verify that it has the same performance in the oral cavity [6–8].

According to our results, PTFE is a good option because it has stability over time, and being a monofilament and smooth, suture could guarantee a lower accumulation of bacterial plaque, thus facilitating a good healing process of soft tissues. However, a combination between the suture material and the technique is not considered to guarantee a better resistance to stress [14, 15, 18]. Finally, in relation to black silk, although it performs well from a mechanical perspective, its main disadvantage is that it also accumulates more bacterial plaque in relation to PG and PTFE [10, 17]. Nonetheless, there are reabsorbable monofilament sutures that accumulate less bacterial plaque. There are also absorbable sutures (Monocryl and Vicryl) to which are added products containing antimicrobial agents (triclosan) with the intention of reducing infection rates [23].

Based on the results of the present study, it does not intend to give a ranking on what material is better, but on the contrary provide, it scientific evidence that can help clinicians to make decisions when choosing the ideal material suture depending on the type and goals of oral surgery that is done.

The main limitation of the present investigation was that although the results of this *in vitro* study favor the use of nonabsorbable sutures such as PTFE (Teflon), there is little literature to support its use in oral surgeries, so more research is needed on this topic. On the other hand, this Teflon suture presented excellent physical properties because its smooth surface favors the nonadherence of bacterial plaque, which added to its excellent traction resistance properties over time, makes it a material of choice. However, many times its availability means that access to it is not mainly due to a cost issue. For this reason, this research proposes and endorses its potential use in oral surgery.

Another of the limitations of this research was that we only work with multifilament suture materials, such as black silk and polyglactin, because some biomaterials of better clinical performance do not reach Latin American countries and are only marketed in developed countries. This in turn increases costs and availability; therefore, it is necessary to create evidence with conventional suture materials. The main reason why it was decided to compare easily accessible sutures such as black silk and polyglactin versus PTFE suture. Finally, the main limitations of this research was the methodological design that only focused on an in vitro study, so it is suggested to conduct longitudinal and clinical studies with a longer evaluation time to confirm the findings found in this study. Certain variables must also be controlled such as suture technique, type of saliva, diet, hygiene habits, and so on because they could directly affect the performance of the suture material.

## 5. Conclusions

This work showed that when comparing the *in vitro* tensile strength of PG, BS, and PTFE sutures at baseline, day 3, day 7, day 14, and day 21, there was no statistically significant difference, *p*=0.870. However, the individual comparison (according to immersion time) showed that the Teflon suture maintains its dimensional stability against tensile forces, which in addition to its biological advantages, allows this material to be considered as an alternative to sutures, even when its availability and cost may influence decision making.

## Figures and Tables

**Table 1:** 

Sodium chloride	0.084 g
Potassium chloride	0.120 g
Calcium chloride dehydrate	0.015 g
Magnesium chloride hexahydrate	0.005 g
Sodium carboxymethylcellulose	0.375 g
Propylene glycol	4.000 g
Methyl paraben	0.100 g
Own paraben	0.010 g
Distilled water csp	100.000 ml

**Table 2:** Tensile strength of various absorbable and nonabsorbable sutures according to immersion time in synthetic saliva.

Suture material	Immersion time	Mean ± SD	Min	Max	*p* ^*∗*^	*p*
PG	Baseline	26.67 ± 3.11	20.02	30.69	0.180	0.522^*∗∗*^
	Day 3	27.55 ± 1.67	25.13	30.20	0.546	
	Day 7	27.01 ± 1.59	22.78	29.76	0.341	
	Day 14	27.49 ± 2.02	6.77	7.92	0.271	
	Day 21	28.14 ± 0.89	6.73	7.49	0.252	

BS	Baseline	14.58 ± 1.64	10.12	15.72	0.001	0.001^*∗∗*^
	Day 3	12.81 ± 2.25	7.75	15.87	0.329	
	Day 7	9.91 ± 9.90	8.54	11.87	0.270	
	Day 14	12.87 ± 0.78	12.28	14.91	0.001	
	Day 21	12.54 ± 0.95	11.48	14.57	0.110	

PTFE	Baseline	7.52 ± 0.94	4.88	8.22	0.001	0.094^*∗∗∗*^
	Day 3	7.59 ± 0.27	7.23	8.10	0.582	
	Day 7	7.84 ± 0.26	7.34	8.21	0.661	
	Day 14	7.48 ± 0.29	6.77	7.92	0.100	
	Day 21	7.21 ± 0.23	6.75	7.49	0.433	

The tensile strength values are means ± standard deviations expressed in Newtons. PG: polyglactin; BS: black silk; PTFE: polytetrafluoroethylene (Teflon). ^*∗*^Shapiro–Wilk normality test; ^*∗∗*^Two-way ANOVA test; ^*∗∗∗*^Kruskal–Wallis test.

## Data Availability

The data used to support the findings of this study are available from the corresponding author upon request.
